# Subregion-based maximum similarity search registration method for transverse chromatic aberration correction in ultra-widefield scanning laser ophthalmoscopy images

**DOI:** 10.1117/1.JBO.31.8.086001

**Published:** 2026-08-01

**Authors:** Lin Ji, Yunshuai Mi, Dewen Xu, Shuo Wang, ShuaiWei Mu, Yun Xiao, HaoMin Yang, Wei Huang, Yunhai Zhang, Wei Xia

**Affiliations:** a University of Science and Technology of China, School of Biomedical Engineering (Suzhou), Division of Life Science and Medicine, Hefei, China; b Suzhou Institute of Biomedical Engineering and Technology, Chinese Academy of Sciences, Suzhou, China; c Key Laboratory of Biomedical Imaging Science and System, Chinese Academy of Sciences; State Key Laboratory of Biomedical Imaging Science and System, Suzhou, China; d The First Affiliated Hospital of Soochow University, Department of Ophthalmology, Suzhou, China

**Keywords:** ultra-widefield scanning laser ophthalmoscopy, transverse chromatic aberration correction, image registration, retinal vessels, perspective transformation

## Abstract

**Significance:**

Ultra-widefield scanning laser ophthalmoscopy (UWF SLO), a confocal scanning-based ophthalmic imaging modality widely used in clinical and research ophthalmology, offers an ultra-wide field of view, high resolution, and real-time dynamic imaging. However, multiwavelength imaging-induced transverse chromatic aberration (TCA) is markedly exacerbated in the peripheral retina, causing ghosting artifacts of retinal vessels and other critical anatomical structures. This severely limits image quality and the reliability of downstream quantitative analysis.

**Aim:**

We aim to correct TCA in multiwavelength UWF SLO images using a subregion-based maximum similarity search registration method, thereby eliminating vascular ghosting artifacts to enhance image quality for reliable clinical analysis.

**Approach:**

In this study, we address the nonuniform TCA between the red and green channels in multiwavelength UWF SLO imaging through three core steps: grid-based subregion sampling, high-precision control point extraction via local optimal matching guided by zero-mean normalized cross-correlation (ZNCC), and global perspective transformation model fitting. The effectiveness of the proposed method was validated on fundus image samples acquired by our in-house-developed UWF SLO system, through both qualitative visual assessment and quantitative evaluation metrics.

**Results:**

The proposed correction method significantly mitigated TCA in UWF SLO images and greatly improved the spatial alignment of retinal vascular contours between the red and green channels. Validation on 11 fundus images showed a significant enhancement in vascular matching performance: the mean dice similarity coefficient increased by 26.7% relatively (from 0.595±0.060 to 0.754±0.035) and the mean intersection over union increased by 42.5% relatively (from 0.426±0.061 to 0.607±0.044). Paired samples t-test verified highly statistically significant improvements in both metrics (all p<0.001).

**Conclusions:**

The proposed subregion-based maximum similarity search registration method effectively corrects TCA in multiwavelength UWF SLO fundus images, significantly improves image quality, and provides reliable technical support for clinical ophthalmic practice and downstream quantitative fundus image analysis.

## 1 Introduction

Ultra-widefield scanning laser ophthalmoscopy (UWF SLO) achieves ultra-widefield fundus imaging via the design of a transmission-type ultra-widefield imaging lens or an ultra-widefield ellipsoidal mirror. According to the consensus definition proposed by the International Widefield Imaging Study Group,^1^ ultra-widefield imaging is defined as retinal imaging that captures the far periphery anterior to the vortex vein ampulla in all four quadrants, corresponding to a retinal imaging angle of ∼110 to 220 deg. It adopts a confocal optical system to detect fundus reflected light signals and generates retinal images with an ultra-large field of view, high spatial resolution, and high contrast.^2^^–^^4^ This modality brings the "hidden zone" of the peripheral retina into the scope of routine clinical observation, significantly improving the early detection rate of lesions, including retinal breaks, retinal degeneration, and peripheral vascular diseases. It thus enables earlier diagnosis, more comprehensive assessment, and more effective disease management of ocular disorders such as diabetic retinopathy.^5^^,^^6^ Therefore, UWF SLO represents not only an expansion of the imaging field of view but also a revolution in the diagnosis and management paradigm of fundus diseases.

In UWF SLO systems, edge artifacts of vascular structures are primarily caused by transverse chromatic aberration (TCA) of multiwavelength lasers, which stems from chromatic dispersion of the ocular refractive media (cornea, lens, vitreous body).^7^^–^^9^ The wavelength-dependent refractive index of ocular media leads to transverse focal displacement on the retina, resulting in misalignment of the same anatomical structure across monochromatic channels and vascular ghosting in the composite color image, which severely hinders accurate segmentation and morphological analysis of fundus vessels. This challenge is particularly severe in ultra-widefield imaging, where the large field of view increases off-axis aberrations and magnifies peripheral TCA. In addition, substantial inter-individual variability in ocular chromatic aberration,^10^ influenced by factors such as retinal eccentricity,^11^ pupil displacement, and residual monochromatic aberrations,^12^ further complicates accurate TCA correction.

Existing TCA correction methods for imaging systems can be broadly classified into optical hardware-based and digital image processing-based strategies. Optical hardware corrections have been extensively explored, including optimized glass selection frameworks based on multi-objective design,^13^ hybrid refractive-diffractive configurations for multichannel imaging,^14^ dispersion-compensated doublet systems enabling broadband apochromatic correction,^15^ and emerging metasurface-integrated gradient-index optics for compact multicolor aberration control.^16^ Collectively, these designs have significantly improved chromatic performance; however, they are fundamentally constrained by fixed optical configurations and cannot accommodate inter-individual variability in ocular aberrations.

**Fig. 1:**
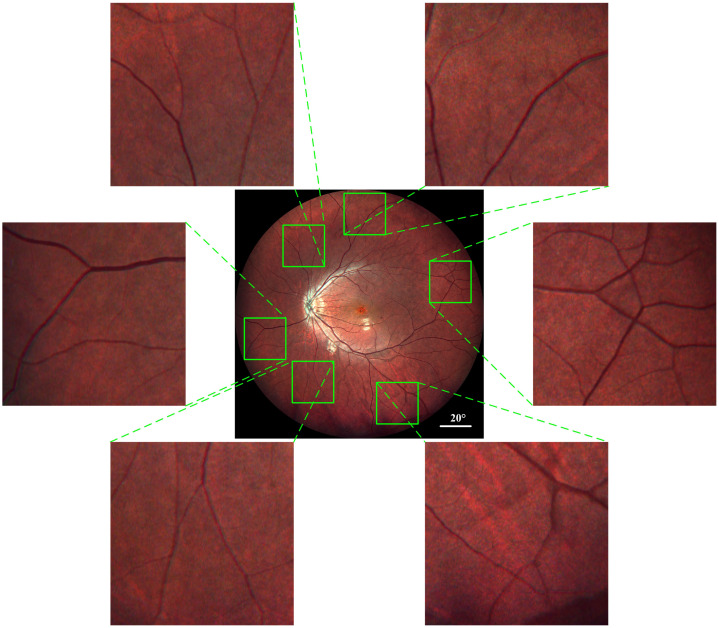
Representative raw multiwavelength fundus images acquired by the in-house-developed UWF SLO system using three laser wavelengths (488, 532, and 638 nm). Red–green channel misalignment of vascular structures in the peripheral retina can be observed in the images.

Figure 1 presents raw multiwavelength fundus images acquired from our in-house UWF SLO system. Red–green channel misalignment of vascular structures in the peripheral retina can be observed, even though we comprehensively suppressed both transverse and longitudinal chromatic aberrations in the optical design through techniques such as apochromatic correction. The results indicate that optical hardware correction alone is insufficient to fully compensate for residual TCA induced by ultra-widefield imaging geometry and subject-specific ocular differences, highlighting the necessity of digital post-processing correction strategies.

In the field of digital image processing, commercial UWF SLO systems (e.g., Optos) are equipped with proprietary algorithms for multiwavelength channel fusion, whereas their detailed implementation procedures and parameters are not publicly available. In the academic literature, Jakstys et al.^17^ proposed TCA correction methods based on a single distortion center and a global transformation model (e.g., perspective or radial), which cannot accommodate the spatially variant aberration that varies with field angle in ultra-widefield fundus images. In adaptive optics scanning laser ophthalmoscopy, measuring and correcting TCA for single-cone stimulation has received considerable attention. Harmening et al.^18^ addressed TCA via interleaved image registration, and Boehm et al.^12^ via pupil tracking with a linear model. Both treat TCA as a field-constant or solely pupil-dependent uniform shift, valid only for small central fields. In UWF SLO, however, TCA varies nonlinearly with eccentricity, making these narrow-field methods inapplicable. Katz et al.^19^ developed a real-time linear TCA correction method based on distortion center calibration. Although effective for their laparoscope, this linear assumption fails for ultra-widefield fundus images where TCA is spatially nonlinear. Legg et al.^20^ applied feature neighborhood mutual information and thin-plate spline (TPS) for cross-modal retinal image registration, but their method does not address TCA correction, and TPS may overfit the smooth radial pattern of TCA. Consequently, these methods often fail to achieve the desired correction in the peripheral retina, where correction is most needed.

By contrast, the method proposed in this paper is specifically tailored for TCA correction in UWF SLO images. It divides the entire circular field of view into grid-based subregions, adopts zero-mean normalized cross-correlation (ZNCC) to achieve robust cross-wavelength matching against illumination fluctuations, and fits a global perspective (homography) transformation with 8 degrees of freedom to simultaneously eliminate minor central shifts and severe peripheral distortions. Requiring no pupil tracking, system pre-calibration, or dedicated hardware, the proposed method can adapt to inter-subject variations in ocular chromatic aberration.

## 2 Materials and Methods

### 2.1 In-House Developed UWF SLO System and Its TCA Characteristics

**Fig. 2:**
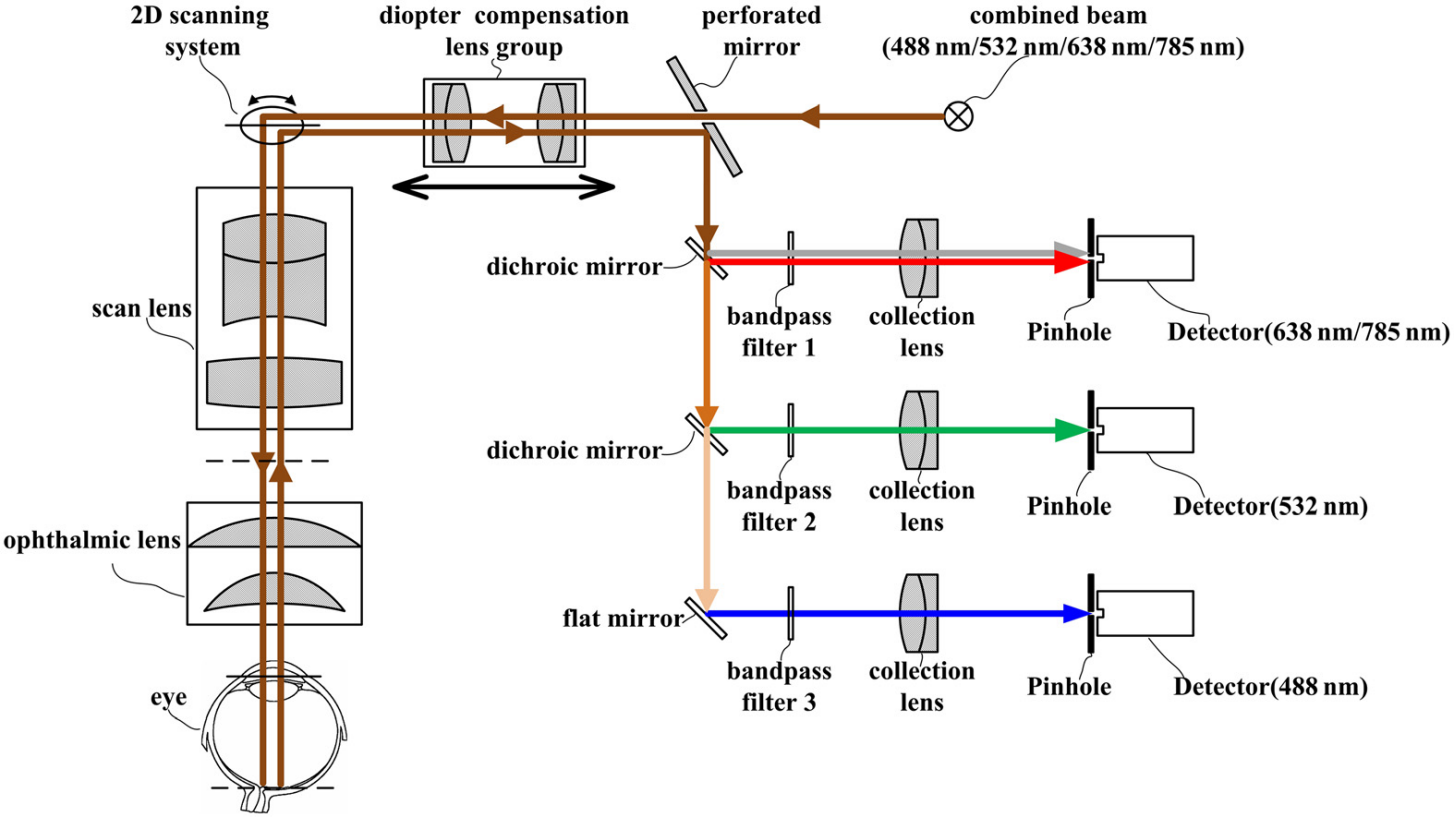
Schematic of the in-house-developed UWF SLO system. A customized multiwavelength laser module internally combines four independently controllable laser sources (488, 532, 638, 785 nm) and couples the combined beam into a single-mode fiber for output, sharing a unified scanning optical path through a perforated mirror, a diopter compensation lens group, a scan lens, and an ophthalmic lens to illuminate the retina. The 785 nm laser provides a real-time preview, whereas the 488, 532, and 638 nm lasers synchronously capture color fundus images in a single scan. In detection, returning light is reflected by the perforated mirror and split by dichroic mirrors into three channels (638/785 nm shared; 532 and 488 nm dedicated), each equipped with a bandpass filter, a collection lens, and a confocal pinhole (1.5 Airy disk diameters).

This study used our in-house UWF SLO system, which employed a customized multi-wavelength laser module that internally combined four independently controllable laser sources (785 nm for preview imaging; 488, 532, and 638 nm for synchronous color fundus acquisition) and coupled the combined beam into a single-mode fiber for output. As shown in Fig. 2, the combined beam passes through a perforated mirror and a diopter compensation lens group, is converted into a 2D scanning beam by the scanning system, and enters the pupil via a scan lens and an ophthalmic lens to illuminate the retina. The beam diameter at the scanning system is 3.9 mm and is demagnified to 1 mm at the pupil plane, corresponding to a reduction factor of ∼3.9×. The returning signal retraces the illumination path and is reflected by the perforated mirror into the detection arm and then split by dichroic mirrors into three detection channels: the 638 and 785 nm signals share one channel, whereas the 532 and 488 nm signals each have a dedicated channel. In each channel, the signal passes through a bandpass filter, a collection lens, and a confocal pinhole (1.5 Airy disk diameters) before reaching the detector. Specifically, the 638 and 785 nm shared channel employs a dichroic mirror (shortpass, cutoff wavelength 605 nm, transmission band 470 to 590 nm, reflection band 620 to 800 nm) combined with a custom multichannel bandpass filter (center wavelengths 638 and 785 nm, FWHM bandwidth 20 nm); the 532 nm channel uses a dichroic mirror (shortpass, cutoff wavelength 505 nm, transmission band 390 to 490 nm, reflection band 520 to 800 nm) with a bandpass filter (center wavelength 532 nm, FWHM bandwidth 10 nm); and the 488 nm channel uses a bandpass filter (center wavelength 488 nm, FWHM bandwidth 10 nm).

The 785 nm laser scans continuously in preview mode to select the imaging region, whereas refractive errors can be adjusted by axially translating the diopter compensation lens group. The system then switches to color acquisition mode: the 785 nm laser is turned off, and the three visible lasers are activated synchronously for a single-frame scan, acquiring red, green, and blue channel data that are fused into a color fundus image. A 125 MHz multichannel DAQ card oversamples each spatial point, and the average of consecutive samples is taken as the final pixel intensity. Preview mode provides 1024×1024  pixels at 7.6 fps; color mode provides 2048×2048  pixels at 3.8 fps. The system achieves a 110 deg scan angle at the pupil (corresponding to a 163 deg retinal imaging angle) and a theoretical lateral resolution of 18  μm. The ophthalmic lens surface features a custom multilayer dielectric coating, which suppresses back-reflections and provides broadband anti-reflection across the 400 to 800 nm spectral range.

For laser safety, the measured corneal incident powers in both preview (785 nm) and color (488/532/638 nm) modes are within safe limits. Assessed according to IEC 60825-1:2014 and ISO 15004-2:2024,^21^^,^^22^ considering retinal thermal and visible-band photochemical hazards, the device falls below the group 1 instrument limits and the class 1 laser product limit. It therefore poses no optical radiation hazard and can be used safely.

Based on the visible spectral absorption characteristics of blood, the green channel (532 nm) provides the highest contrast between retinal vessels and background tissue, thereby offering the clearest visualization of vascular contours and fine structural details.^23^^,^^24^ Therefore, the green channel was selected as the fixed reference benchmark for the subsequent registration and correction procedures.

In multiwavelength UWF SLO imaging, TCA arises from the wavelength-dependent refractive index of the ocular media. The TCA between the blue (488 nm) and green (532 nm) channels is negligible due to their closely spaced wavelengths. By contrast, the red channel (638 nm), exhibiting the largest wavelength separation from the reference green channel, constitutes the primary source of TCA in color imaging. Furthermore, owing to the eye’s optical geometry and curved fundus, TCA is spatially nonlinear—minimal centrally, progressively severe peripherally—rather than a simple global shift. Correcting this spatially varying displacement is the central challenge in UWF fundus imaging.

### 2.2 Subregion-Based Maximum Similarity Search Registration for TCA Correction

**Fig. 3:**
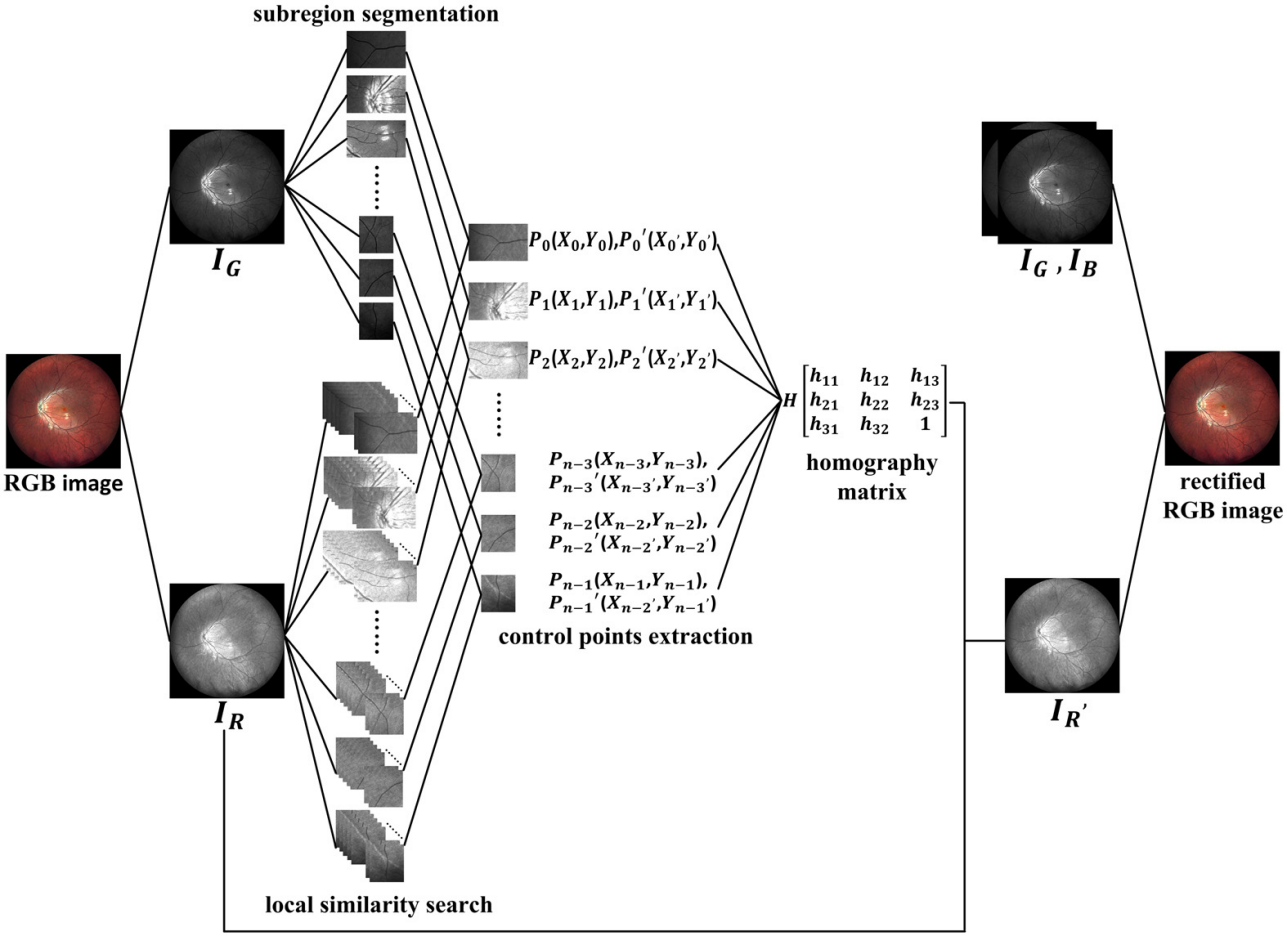
Workflow of the proposed subregion-based channel registration method for TCA correction. The process comprises five main steps: (1) image channel separation and reference channel selection, (2) subregion segmentation, (3) control point extraction via local similarity search, (4) global homography matrix estimation, and (5) red channel spatial correction and image reconstruction.

To address the challenge of nonuniform TCA correction in multiwavelength UWF SLO images, this study proposes a channel registration method based on a subregion-based maximum similarity search. Using the high-contrast green channel as a fixed reference, the method extracts high-precision inter-channel control points through subregion segmentation and local similarity search. A global homography transformation model is then estimated and applied to the red channel with spatial resampling, achieving accurate red-green channel registration and full-field TCA correction. As illustrated in Fig. 3, the core workflow comprises five steps: (1) image channel separation and reference channel selection, (2) subregion segmentation, (3) control points extraction via local similarity search, (4) global homography matrix estimation, and (5) red channel spatial correction and image reconstruction.

#### 2.2.1 Image channel separation and subregion segmentation

Although our in-house developed UWF SLO records each wavelength channel independently and could skip channel separation, we include this step to ensure compatibility with commercial UWF SLO devices that typically provide only composite RGB images. Accordingly, the workflow below starts with channel separation.

In our implementation, channel separation extracts the green ($I_{G}$) and red ($I_{R}$) channel images from the composite color fundus image. $I_{G}$, with high vessel contrast, is set as the fixed reference; $I_{R}$ serves as the target. The objective is to warp $I_{R}$ into the coordinate space of $I_{G}$ to eliminate inter-channel TCA.

Given the nonuniform TCA distribution in multiwavelength UWF SLO images, a single global registration cannot achieve consistent correction accuracy across central and peripheral fields. We therefore adopted a grid-based division strategy, partitioning the reference image $I_{G}$ into discrete full-coverage subregions to accurately capture region-specific TCA offsets through local sampling.

**Fig. 4:**
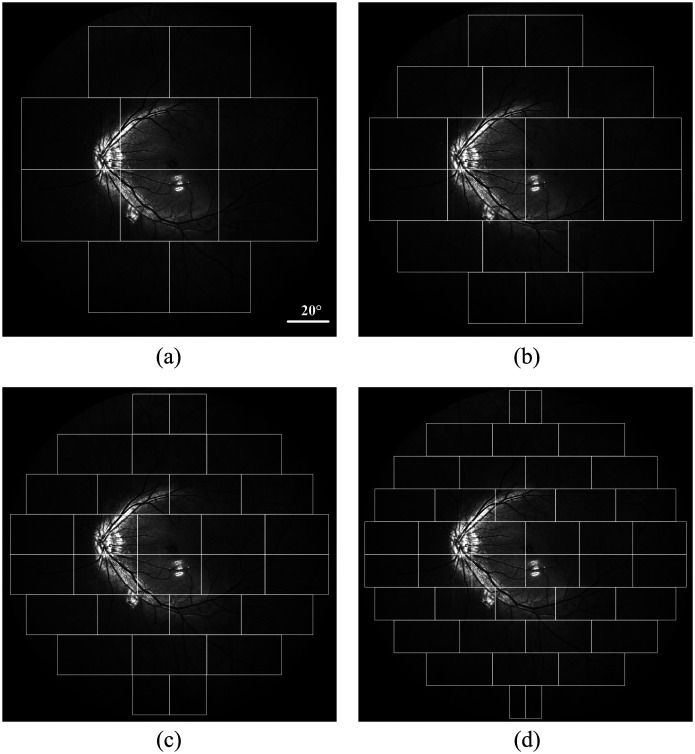
Representative subregion segmentation methods. (a) Two-level segmentation. (b) Three-level segmentation. (c) Four-level segmentation. (d) Five-level segmentation. The subregion count trades off correction accuracy against computational cost and mismatch risk. The four-level scheme (c) is adopted for its favorable balance between peripheral TCA correction and manageable overhead.

Figure 4 illustrates several typical subregion segmentation methods. We selected the four-level segmentation scheme [Fig. 4(c)], which divides the reference image into 28 subregions. This configuration balances sampling density in the central field with full coverage of the peripheral field, enabling effective capture of nonlinear TCA distortion in ultra-widefield marginal areas.

The number of subregions is determined by a fundamental trade-off: more subregions improve local sampling density and correction accuracy but increase computational load and the risk of mismatching; conversely, too few subregions fail to model nonuniform TCA, resulting in insufficient peripheral correction. In addition, the number of subregions must be at least four to support the subsequent solution of the global homography transformation matrix. This issue is further discussed in Sec. 2.4, which investigates the influence of subregion partition levels on algorithm performance.

#### 2.2.2 ZNCC-based local similarity search: extracting matched control points

For each subregion in the reference green channel, we performed an exhaustive search within the corresponding local neighborhood in the red channel, identifying the optimal matching region using a similarity metric to construct matched control point pairs. This step is fundamental to achieving local fine registration.

The specific implementation workflow is as follows: for the i’th subregion in the reference image $I_{G}$, we recorded its center coordinates $P_{i^{\prime }}(X_{i^{\prime }},Y_{i^{\prime }})$, and determined its initial corresponding position in the target red channel image $I_{R}$, centered at these coordinates. Using this initial position as the center, we defined a search radius R=10  pixels (image size 2048×2048)—empirically determined to accommodate the maximum chromatic aberration-induced offset of our system—and performed a two-dimensional exhaustive traversal within this circular neighborhood. The search was conducted in both horizontal and vertical directions with a step size of 1 pixel, generating a series of candidate matching regions of the same dimensions as the reference subregion.

Common image registration similarity metrics include sum of absolute differences (SAD) and sum of squared differences (SSD), which are based on pixel intensity differences, as well as zero-mean normalized cross-correlation (ZNCC), which is based on statistical correlation.^25^ In multiwavelength images, channels exhibit global intensity differences from varying laser power and detector responses. SAD and SSD are sensitive to such variations and prone to mismatches.^26^ ZNCC, by subtracting the mean and normalizing, achieves invariance to linear intensity changes, effectively compensating for inter-channel differences and providing robust cross-channel matching.^27^ We therefore adopt ZNCC to measure similarity between candidate regions and the reference subregion, select the candidate with the maximum ZNCC as the optimal match, and record its center coordinates $P_{i}(X_{i},Y_{i})$.

For two image patches I (reference subregion) and J (candidate matching region), both of size M×N, the ZNCC calculation formula is

$$\mathrm{ZNCC}(I,J)=\frac{\sum_{x=1}^{M}\sum_{y=1}^{N}[I(x,y)-\mu_{I}][J(x,y)-\mu_{J}]}{\sqrt{\sum_{x=1}^{M}\sum_{y=1}^{N}[I(x,y)-\mu_{I}{]}^{2}\cdot \sum_{x=1}^{M}\sum_{y=1}^{N}[J(x,y)-\mu_{J}{]}^{2}}},$$ (1)

where I(x,y) and J(x,y) denote the pixel intensity values of image patches I and J at coordinates (x,y), respectively; $\mu_{I}$ and $\mu_{J}$ are the mean pixel intensities of I and J, respectively, satisfying $\mu_{I}=\frac{1}{M\cdot N}\underset{x,y}{\sum }I(x,y)$ and $\mu_{J}=\frac{1}{M\cdot N}\underset{x,y}{\sum }J(x,y);M$ and N are the width and height of the image patches (in pixels), respectively.

Finally, the center coordinates of the reference subregion, denoted as $P_{i^{\prime }}(X_{i^{\prime }},Y_{i^{\prime }})$, and the center coordinates of its corresponding optimal matching region in the red channel, denoted as $P_{i}(X_{i},Y_{i})$, are paired to construct a discrete set of matched control point pairs ${\left\{P_{i},P_{i^{\prime }}\right\}}_{i=0}^{n-1}$, where n is the total number of control point pairs.

#### 2.2.3 Global perspective transformation (homography) model and matrix estimation

Global 2D translation models (e.g., Guizar-Sicairos et al.^28^) assume a rigid shift and cannot capture the nonuniform, periphery-enhanced TCA in UWF SLO images. The affine model adds rotation, scaling, and shear but preserves line parallelism, which limits it to linear distortions and prevents modeling the progressive peripheral nonlinearity.^29^ We therefore adopt the perspective transformation (homography), which relaxes the parallelism constraint, models the 3D-to-2D projection, and uses eight parameters to simultaneously correct the small central offsets and the large peripheral distortions.

Perspective transformation maps pixel coordinates via homogeneous coordinates using a 3×3 matrix H with 8 degrees of freedom, expressed as follows:

$$[\begin{array}{c} X_{i}^{\prime }\\ Y_{i}^{\prime }\\ 1 \end{array}]=H[\begin{array}{c} X_{i}\\ Y_{i}\\ 1 \end{array}]=[\begin{array}{ccc} h_{11} & h_{12} & h_{13}\\ h_{21} & h_{22} & h_{23}\\ h_{31} & h_{32} & 1 \end{array}][\begin{array}{c} X_{i}\\ Y_{i}\\ 1 \end{array}],$$ (2)

where $\left(X_{i},Y_{i}\right)$ is the pixel coordinate of the original red channel image before correction and $\left({X_{i}}^{\prime },{Y_{i}}^{\prime }\right)$ is the corresponding matching point coordinate in the reference green channel. From each matched pair, eliminating the scale factor yields two linearly independent equations:

$$\left\{ \begin{array}{l} X_{i}h_{11}+Y_{i}h_{12}+h_{13}-X_{i}X_{i^{\prime }}h_{31}-Y_{i}X_{i^{\prime }}h_{32}=X_{i^{\prime }}\\ X_{i}h_{21}+Y_{i}h_{22}+h_{23}-X_{i}Y_{i^{\prime }}h_{31}-Y_{i}Y_{i^{\prime }}h_{32}=Y_{i^{\prime }} \end{array}.\right.$$ (3)

At least four noncollinear point pairs are required to solve for H. In practice, full-field grid-based subregion division provided abundant high-precision control point pairs—far exceeding this minimum. After removing outliers with RANSAC (2-pixel reprojection error, 2000 iterations, 0.995 confidence), we constructed an overdetermined linear system via the direct linear transform and solved it by singular value decomposition to obtain the globally optimal H that minimizes the algebraic reprojection error. The entire estimation was implemented using the “findHomography” function in the OpenCV library.

We applied forward spatial mapping to the red channel using H and resampled the resulting noninteger coordinates via bilinear interpolation to reduce aliasing, producing a red channel aligned with the green reference. Replacing the original red channel with this corrected version yielded the final TCA-corrected color fundus image, completing the workflow.

### 2.3 Correction Effect Validation Scheme

To comprehensively and objectively evaluate the performance of the proposed TCA correction method, we established two complementary validation schemes covering qualitative visual assessment and quantitative performance evaluation. The qualitative validation focused on subjective visual effect assessment, whereas the quantitative validation was based on objective metrics. All validation experiments were conducted on 11 ultra-widefield color fundus image samples acquired by our in-house-developed UWF SLO system. The 11 subjects comprised seven males and four females, aged 18 to 56 years, with myopia, hyperopia, and normal vision represented; for each subject, one eye was randomly selected (left or right).

#### 2.3.1 Qualitative visual validation

To intuitively demonstrate the performance difference before and after TCA correction, we adopted a two-tiered visual comparison scheme comprising the following components:

a. Validation of local details and intensity distribution in original channel images

We compared magnified views of corresponding regions from the red and green channel images before and after correction to visually assess changes in the spatial alignment of vascular contours. A fixed measurement line was drawn along the radial direction of a representative vessel, and the normalized intensity distribution curves of the green and red channels along this line were extracted before and after correction. This allowed us to analyze the reduction in transverse signal misalignment within the vascular region.

b. Channel overlay visualization based on vessel segmentation

Using unified fundus vessel segmentation results, we constructed two RGB composite images for overlay comparison before and after correction. In the first image (pre-correction), the red channel was assigned the vessel segmentation result from the uncorrected red channel, whereas the green channel was assigned the segmentation result from the green reference channel. In the second image (post-correction), the red channel was assigned the segmentation result from the corrected red channel, with the green channel remaining unchanged. The blue channels of both images were set to zero. By comparing the proportion of red-green separated regions versus overlapped yellow regions between the two images, we could intuitively visualize the improvement in vascular spatial alignment achieved by the proposed correction method.

#### 2.3.2 Quantitative evaluation metrics

To objectively evaluate the spatial alignment of vascular structures between the red and green channels before and after TCA correction, we used the vessel segmentation results from the green channel as the reference standard. Quantitative assessment was performed using standardized metrics and statistical analysis, as detailed below.

a. Standardized fundus vessel segmentation

It is important to note that the vessel segmentation model (U-Net) introduced here serves exclusively as an independent evaluation tool for generating binary masks to compute quantitative metrics. It is not a component of the proposed TCA correction pipeline described in Sec. 2.2 and plays no role in the correction algorithm itself.

Automated retinal vessel segmentation was performed using the classic standard U-Net fully convolutional neural network architecture. This architecture consists of a symmetric encoder–decoder structure with cross-layer skip connections, which can accurately capture the multiscale features of retinal vessels and achieve pixel-level dense prediction. The model was pre-trained on three publicly available retinal vessel datasets, namely, DRIVE, HRF, and CHASE_DB1, to fully ensure the generalization capability of the model and the reliability of the segmentation results.

To completely eliminate the interference of the model itself on the quantitative evaluation results and ensure the objectivity and uniqueness of the assessment of TCA correction efficacy, fixed pre-trained model weights were used throughout the entire segmentation process for all images to be segmented in this study, without any additional fine-tuning, transfer learning, or domain adaptation performed on the ultra-widefield fundus images involved in this research.

During the segmentation inference procedure, a completely consistent preprocessing pipeline, model parameters, and forward inference protocol were applied to the green channel reference images, pre-correction red channel images, and post-correction red channel images. Specifically, all input images were first uniformly resized to a fixed dimension of 2048×2048  pixels, followed by min–max normalization of the single-channel grayscale values to the range of [0, 1] before being fed into the model. After the model output the vessel probability map, a fixed confidence threshold of 0.5 was applied for binarization, without any additional morphological post-processing operations throughout the entire workflow.

Through the above fully consistent standardized procedure, the reference vessel segmentation result set G, the pre-correction vessel segmentation result set $R_{\mathrm{pre}}$, and the post-correction vessel segmentation result set $R_{\text{post}}$ were finally obtained, respectively, for subsequent quantitative evaluation.

b. Core quantitative evaluation metrics

The dice similarity coefficient (DSC) and intersection over union (IoU) were selected as primary metrics to quantitatively assess the spatial overlap between the red and green channel vessel segmentations. Both metrics range from 0 to 1, with higher values indicating better alignment of vascular structures and, consequently, more effective TCA correction. We selected these mask-based metrics because our primary objective is to correct TCA-induced spatial misalignment of anatomical structures, which DSC and IoU directly quantify. We acknowledge that these metrics measure vascular overlap rather than pixel-wise raw image identity; however, the standardized and fixed segmentation pipeline applied uniformly to all images ensures that the observed relative improvement is attributable solely to improved spatial registration. Their calculation formulas are given in Eqs. (4) and (5), respectively:

$$\mathrm{DSC}=\frac{2|G\cap R|}{\left|G|+|R\right|},$$ (4)

$$\mathrm{IoU}=\frac{\left|G\cap R\right|}{\left|G\cup R\right|},$$ (5)

where |·| denotes the total number of pixels in the vessel region.

c. Statistical analysis

The Shapiro–Wilk test was used to assess the normality of the differences in DSC and IoU values before and after correction. If the differences followed a normal distribution (p>0.05), a paired-sample t-test was applied to compare pre- and post-correction metrics, thereby evaluating the statistical significance of the correction effect. The significance level was set at α=0.05, with p<0.05 considered statistically significant.

### 2.4 Effect of Subregion Partition Levels on Algorithm Performance

The subregion count involves a trade-off between correction accuracy and computational cost. More subregions improve local sampling and correction precision, but the additional control points linearly increase the overhead of RANSAC outlier rejection and global homography estimation. Too few subregions, however, fail to capture the nonlinear peripheral distortion. As the per-subregion ZNCC similarity search cost is nearly independent of the subregion count, the overall computational load is dominated by these post-processing steps.

To evaluate the effect of subregion partition level on performance, we compare four configurations (Fig. 4): level 2 (10 subregions), level 3 (18 subregions), level 4 (28 subregions), and level 5 (40 subregions). All experiments use the following settings.

a. Input images are 2048×2048  pixels; subregion partitioning is restricted to the circular field of view (diameter 2048 pixels).
b. Local similarity search uses a circular neighborhood of radius 10 pixels (Sec. 2.2.2).
c. Computational overhead is measured as average processing time per image on a desktop CPU (3.7 GHz, 16 GB RAM).
d. Mismatch ratio is the percentage of subregions classified as outliers by RANSAC. RANSAC parameters: reprojection error threshold 2 pixels, maximum 2000 iterations, inlier confidence 0.995. This metric is approximate—a small number of erroneous ZNCC matches may coincidentally satisfy the homography and be retained as inliers.
e. Correction accuracy is evaluated using DSC metrics on corrected images from the same 11 subjects (Sec. 2.3).

The measured experimental results (averaged over 11 image samples) are summarized in Table 1.

**Table 1:** Comparison of algorithm performance for various subregion partition levels.

Subregion partition levels	Number of subregions	Local sampling density (per 500 × 500 pixels)	Mismatch percentage (%)	Correction accuracy (Mean ± SD)	Average runtime(s)
2-level	10	0.759	10.9	0.681±0.007	3.76
3-level	18	1.366	12.1	0.720±0.012	4.08
4-level	28	2.125	15.6	0.754±0.035	4.58
5-level	40	3.036	20.0	0.760±0.014	5.65

SD values are reported as sample standard deviation (n-1).

As listed in Table 1, correction accuracy improves markedly from levels 2 to 4 but shows only marginal gain from levels 4 to 5, indicating that 28 subregions adequately model the nonuniform TCA. The mismatch ratio increases with subregion number, reaching 20.0% at level 5 (1.28× level 4), which shows that excessively fine partitioning raises the risk of false matches. Runtime grows monotonically with the subregion count; level 5 yields negligible accuracy improvement yet substantially higher computational cost. Balancing accuracy, mismatch ratio, and efficiency, we select the level-4 configuration (28 subregions) as the default. This ensures sufficient local sampling to capture peripheral nonuniform TCA while maintaining an acceptable mismatch ratio and moderate computational overhead.

## 3 Results

### 3.1 Qualitative Visualization Results

**Fig. 5:**
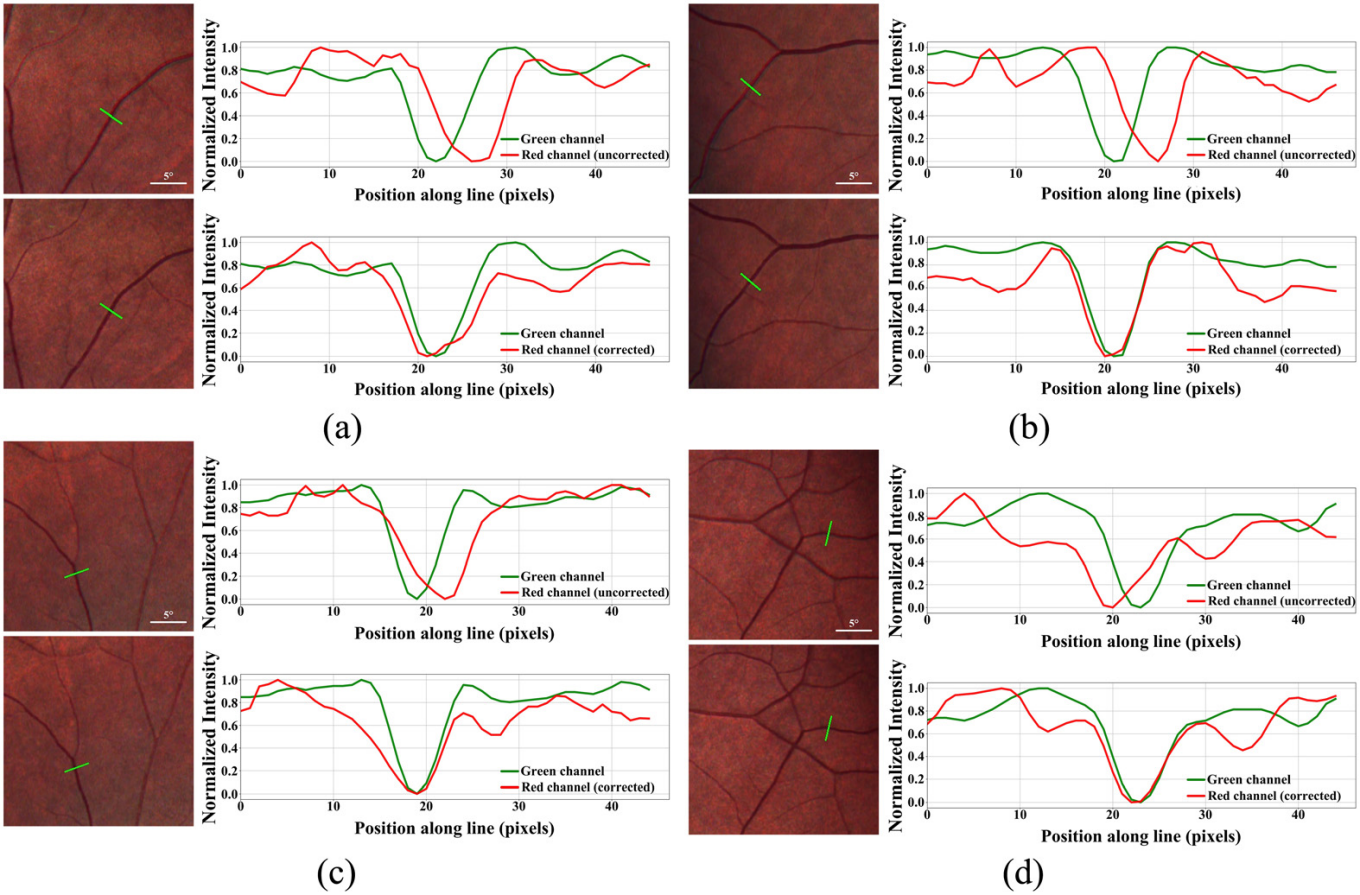
Qualitative comparison of magnified local views and normalized intensity profiles before (upper) and after (lower) TCA correction. Before correction, vascular contours exhibit obvious spatial misalignment, and the intensity profiles show a clear transverse offset. After correction, vascular alignment and profile overlap are markedly improved, confirming effective correction of transverse chromatic aberration. The magnified local views were taken from the peripheral retina at retinal eccentricities of approximately 65.7 deg (a), 63.9 deg (b), 57.5 deg (c), and 63.0 deg (d), respectively.

The proposed subregion-based maximum similarity search registration method was applied to multiwavelength fundus images acquired by our in-house-developed UWF SLO system for TCA correction. As shown in Fig. 5, comparison of local magnified views from representative samples reveals distinct spatial misalignment between the vascular contours of the red and green channels prior to correction. Following correction, the spatial alignment of vascular structures between the two channels is markedly improved. These magnified local views were selected from the peripheral retina, with retinal eccentricities of ∼65.7  deg (a), 63.9 deg (b), 57.5 deg (c), and 63.0 deg (d), respectively, where transverse chromatic aberration is most pronounced.

To quantitatively illustrate this improvement, a measurement line was drawn along the radial direction of a representative vessel, and the normalized intensity distribution curves of the green and red channels along this line were extracted. The results show an obvious transverse offset between the two curves in the vascular region before correction, whereas the overlap between the curves is substantially enhanced after correction.

These qualitative findings demonstrate that the proposed method effectively corrects transverse chromatic aberration in ultra-widefield fundus images, significantly improving the spatial alignment of vascular structures across multiwavelength channels.

### 3.2 Visual Comparison of Vessel Segmentation Results

**Fig. 6:**
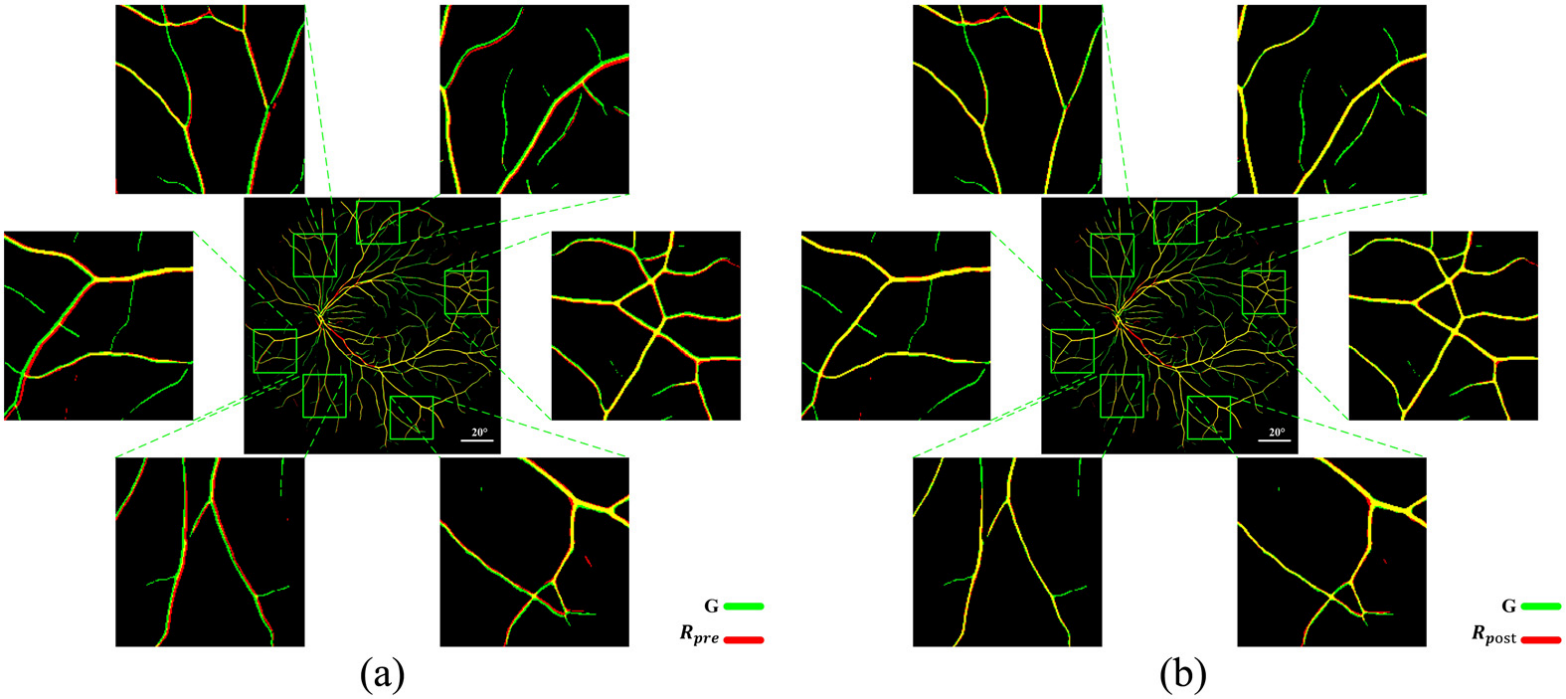
RGB overlay comparison of vessel segmentation results before and after TCA correction for representative samples. Overlays were generated by mapping red-channel vessel masks to the red channel and green-channel vessel masks to the green channel. (a) Pre-correction: separated red and green regions around vessels indicate significant spatial misalignment. (b) Post-correction: separated regions are markedly reduced, and yellow regions (indicating red–green superposition) increase substantially, demonstrating improved spatial overlap.

Vessel segmentation was performed on the three types of images using the U-Net model, and two sets of RGB visualization comparison images before and after correction were generated according to predefined rules (Fig. 6). The results showed that: in the pre-correction RGB images, numerous separated red and green regions were visible around the vessels, indicating that there was marked spatial misalignment between the vessels in the red channel and the green reference channel before correction; in the post-correction RGB images, the separated red and green regions were significantly reduced, and the yellow regions formed by the superposition of red and green channels were greatly increased, intuitively demonstrating that the spatial overlap of vessels between the two channels was significantly improved after correction.

### 3.3 Quantitative Evaluation of TCA Correction

Using the vessel segmentation results of the green channel as the reference standard, we calculated the DSC and IoU values for the red channel before and after correction across 11 ultra-widefield fundus images. The quantitative results are summarized in Table 2. The data show that the vascular alignment metrics between the red channel and the green reference channel improved substantially after applying the proposed correction method: the mean DSC increased from 0.595±0.060 before correction to 0.754±0.035 after correction, representing a relative improvement of ∼26.7%; the mean IoU increased from 0.426±0.061 to 0.607±0.044, corresponding to a relative improvement of ∼42.5%.

**Table 2:** Quantitative statistical results of vascular overlap between red and green channels of fundus images before and after TCA correction.

Subject ID	DSC		IoU	
	Pre	Post	Pre	Post
1	0.574	0.748	0.402	0.598
2	0.613	0.777	0.442	0.635
3	0.556	0.787	0.385	0.649
4	0.678	0.773	0.513	0.629
5	0.578	0.758	0.406	0.610
6	0.556	0.681	0.385	0.516
7	0.550	0.712	0.379	0.553
8	0.482	0.721	0.318	0.564
9	0.632	0.774	0.462	0.631
10	0.656	0.790	0.488	0.653
11	0.669	0.776	0.502	0.635

Each subject ID represents a unique individual; one eye was randomly selected per subject for analysis (11 subjects: 7 males, 4 females; age 18 to 56 years; including myopia, hyperopia, and normal vision). All SD values are reported as sample standard deviation (n-1).

### 3.4 Statistical Analysis

The Shapiro–Wilk normality test confirmed that the pre- to post-correction differences followed a normal distribution for both DSC (W=0.9458,p=0.5915) and IoU (W=0.9538,p=0.6929), with (p>0.05) in both cases (Table 3). Paired-sample t-tests revealed that the post-correction DSC values were significantly higher than those before correction (t(10)=11.49,p<0.001), and the same held true for IoU (t(10)=12.95,p<0.001). These results demonstrate that the TCA correction achieved by the proposed method is statistically significant.

**Table 3:** Statistical test results of correction efficacy.

Metric	DSC			IoU		
	Pre	Post	Difference (Post − Pre)	Pre	Post	Difference (Post − Pre)
Mean	0.595	0.754	0.159	0.426	0.607	0.181
Standard deviation (SD)	0.060	0.035	0.046	0.061	0.044	0.046
Shapiro-Wilk W	—	—	0.9458	—	—	0.9538
Shapiro-Wilk p	—	—	0.5915	—	—	0.6929
Paired t-test t	—	—	11.49	—	—	12.95
Paired t-test df	—	—	10	—	—	10
Paired t-test p (Two-tailed)	—	—	<0.001	—	—	<0.001

SD values are reported as sample standard deviation (n-1).

## 4 Discussion and Conclusions

To correct TCA in UWF SLO images, this study proposes a subregion-based maximum similarity search method. Using the green channel as reference, the image is divided into subregions via a grid, and for each subregion, a ZNCC-based similarity search within the corresponding local neighborhood of the red channel automatically extracts high-precision control point pairs. These point pairs then determine a global perspective transformation that warps the red channel into alignment with the green channel.

Experimental results confirm effective correction both qualitatively and quantitatively. Qualitatively, corrected images show markedly improved spatial alignment of red–green vascular contours, with overlay visualization revealing increased vessel overlap (yellow) and substantially reduced red–green separation. Quantitatively, across 11 UWF SLO images, the mean DSC between red and green vessel segmentations rose from 0.595±0.060 to 0.754±0.035 (≈26.7% improvement), and mean IoU from 0.426±0.061 to 0.607±0.044 (≈42.5% improvement); both gains are statistically significant (paired t-test, p<0.001).

The proposed method requires no additional hardware calibration or prior TCA modeling. By establishing the spatial transformation directly from the image data itself through local adaptive search, it abandons the constraints of fixed transformation models and adaptively solves for the optimal transformation based on the actual image content. This approach not only overcomes the interference of global intensity differences between channels but also flexibly accommodates both inter-individual variations in ocular chromatic aberration and the nonuniform TCA distribution inherent to ultra-widefield imaging.

The proposed method has several limitations. Control point extraction accuracy depends on subregion-based similarity matching, with a risk of mismatches in low-SNR or poorly defined vascular regions. The grid division level entails a trade-off between sampling density and computational cost; although the four-level scheme (28 subregions) performed well, the optimal level may need re-tuning for different imaging systems or diseased retinas. Moreover, TCA correction was evaluated only via vessel segmentation, without assessing the impact on downstream clinical quantitative tasks such as vessel density or lesion area measurement.

Future work will focus on the following directions: first, exploring adaptive grid partitioning strategies that dynamically adjust the size and distribution of subregions based on local image characteristics to further improve correction accuracy; second, introducing deep learning techniques to investigate the feasibility of end-to-end chromatic aberration correction methods, thereby reducing reliance on explicit control point extraction; third, applying the proposed method to large-scale clinical cohort studies to evaluate its clinical value for quantitative analysis of fundus diseases such as diabetic retinopathy and age-related macular degeneration; fourth, exploring the applicability of the method to other multiwavelength imaging systems; and fifth, systematically investigate the influence of pupil offset on TCA in UWF SLO images, and explore the implementation approach for integrating pupil tracking information into the image registration framework.

In summary, the proposed registration method based on subregion-level maximum similarity search effectively corrects transverse chromatic aberration in UWF SLO images and significantly improves the spatial alignment accuracy of vascular structures between the red and green channels. This method offers an effective technical solution for enhancing the quality of multiwavelength fundus imaging and ensuring the reliability of subsequent clinical quantitative analysis.

## Data Availability

The data that support the findings of this article are not publicly available due to privacy restrictions. They may be requested from the corresponding author (zhangyh@sibet.ac.cn). The code supporting the findings of this study will be made publicly available on GitHub (https://github.com/jilinhfut88/SBMSSR.git) upon acceptance of the paper.
